# Prevalence and Nature of Financial Considerations Documented in Narrative Clinical Records in Intensive Care Units

**DOI:** 10.1001/jamanetworkopen.2018.4178

**Published:** 2018-11-02

**Authors:** Deborah D. Gordon, Ihsaan Patel, Amelia M. Pellegrini, Roy H. Perlis

**Affiliations:** 1 Mossavar-Rahmani Center for Business and Government, Harvard Kennedy School, Cambridge, Massachusetts; 2 Center for Quantitative Health, Division of Clinical Research and Center for Genomic Medicine, Massachusetts General Hospital, Boston; 3 Department of Psychiatry, Harvard Medical School, Boston, Massachusetts

## Abstract

**Importance:**

The extent to which financial considerations alter intensive care unit (ICU) decision making is poorly understood.

**Objectives:**

To characterize the prevalence and nature of financial considerations documented in narrative clinical records and their association with patient-level demographic and clinical features.

**Design, Setting, and Participants:**

In silico cohort study applying natural language processing to narrative notes from the Medical Information Mart for Intensive Care (MIMIC-III) study. Data from all individuals hospitalized between June 1, 2001, and October 31, 2012, in the ICU of Beth Israel Deaconess Medical Center were analyzed from April 1 to April 30, 2018.

**Main Outcomes and Measure:**

Presence of financial considerations in narrative clinical notes.

**Results:**

Among 46 146 index ICU admissions, 1936 patients (4.2%) were identified with at least 1 note reflecting financial considerations during the ICU stay. Of these 1936 patients, 1135 (58.6%) were male, with a mean (SD) age of 38.8 (28.4) years and mean (SD) length of stay of 21.7 (27.1) days. Among the remaining 44 210 admissions in the cohort, 24 780 (56.1%) were male, with a mean (SD) age of 48.6 (32.1) years and mean (SD) length of stay of 9.2 (11.4) days. Among the 46 146 admissions, 142 (0.3%) included notes describing a change in the discharge plan, 142 (0.3%) describing a change in the treatment plan, and 303 (0.7%) describing a change in medication or previous nonadherence to medication associated with financial considerations. In logistic regression models adjusted for age, sex, marital status, and insurance type, longer hospital stays were significantly associated with the presence of financial notes (odds ratio, 1.01; 95% CI, 1.01-1.01).

**Conclusions and Relevance:**

In this study, among patients in the ICU, clinical notes document the association of financial considerations with care decisions. Although such notes likely underestimate the frequency of such considerations, they highlight the need to develop better systematic approaches to understanding how financial constraints may alter care decisions in US health systems.

## Key Points

**Question:**

How often are cost considerations documented in narrative clinical notes, and do those considerations influence treatment decisions?

**Findings:**

In an in silico cohort study of narrative clinical notes from 46 146 index admissions to the intensive care unit at a large academic medical center, 4% had at least 1 note reflecting financial considerations during the intensive care unit stay.

**Meaning:**

Even in the intensive care unit setting, financial considerations are addressed and may be associated with adjustments to treatment decisions.

## Teaser

This in silico cohort study uses data from the Medical Information Mart for Intensive Care (MIMIC-III) to investigate whether discussions documented in narrative clinical notes about financial concerns between patients and clinicians are associated with health care decision making in the intensive care unit.

## Introduction

The role of a market economy in controlling health care costs has been the subject of major debate, ranging from whether such a market can control costs to whether it can exist in the first place.^1,2,3^ Particularly unclear is the extent to which clinicians and patients participate in such a market, that is, how much financial considerations may alter decisions about clinical care at the level of individual patients and clinicians. Clinicians often do not have the knowledge or processes established to provide cost information or effectively engage in cost discussions with patients. An analysis of transcripts of more than 1700 community clinic visits suggested that as many as 1 in 3 visits included discussion of cost.^4^

Furthermore, many clinicians have been trained not to consider financial issues when making treatment decisions, although survey results suggest that such considerations may be common, at least in oncology.^5,6^ Professional societies have advocated for consideration of cost^7^; and cost considerations are addressed in some medical school curricula.^8^ The extent to which such considerations are addressed in practice and alter treatment decisions is difficult to quantify because these processes are not typically documented in the large clinical data sets most widely available for study, such as private and public payer claims databases.

Electronic health records may provide an alternative means of understanding real-world decision-making processes. With the availability of machine learning methods to efficiently capture features in narrative clinical notes, it is possible to define concepts that might not otherwise be available for study at scale.^9^ These methods have been applied successfully to a range of clinical problems, which are part of a transformative wave of artificial intelligence studies in medicine. However, to our knowledge, they have not yet been applied to understanding the role of financial considerations in health care decisions.

To investigate how financial concerns are associated with health care decision making, we developed a set of classifiers to identify the presence and nature of such concerns in clinical documentation. We sought to understand not only the prevalence of these topics but the extent to which they might be associated with patient-level sociodemographic features.

## Methods

### Study Design

For the present study, we used a large corpus of narrative clinical notes drawn from the Medical Information Mart for Intensive Care (MIMIC) (MIMIC-III, version 1.4),^10^ which includes deidentified clinical documentation for a cohort of 46 146 individuals treated in the intensive care units (ICUs) of Beth Israel Deaconess Medical Center in Boston, Massachusetts, a large academic medical center, between June 1, 2001, and October 31, 2012. No other inclusion or exclusion criteria were applied. The data were accessed via a data use agreement between PhysioNet, a National Institutes of Health–supported data repository (https://www.physionet.org/), and one of us (I.P.), dated May 18, 2017, and analyzed from April 1 to April 30, 2018. Only the index admission for each patient was considered in the primary analyses because we hypothesized that multiple ICU admissions might be more likely to precipitate financial conversations and render results among all ICU admissions less interpretable (ie, if they reflect many admissions for a small number of patients). This study followed the Strengthening the Reporting of Observational Studies in Epidemiology (STROBE) reporting guideline. This study was approved by the institutional review board of Massachusetts General Hospital, which also waived the need for patient informed consent because the data were deidentified.

First, to maximize sensitivity, a set of intentionally broad seed terms reflecting financial concepts was manually curated by 2 of us (D.D.G. and I.P.) because no applicable lexicon could be identified. Seed terms included *cost, insurance, pay, afford, financial, expensive, expense, out of pocket, Medicare*, and derivatives of these. Exact string matching was used to identify putative financial notes, yielding 5238 such notes from a total of 2 083 180 narrative notes that compose MIMIC-III. All of these 5238 notes were manually reviewed by one of us (D.D.G.) to confirm documentation of financial considerations relevant to clinical care, yielding 3302 notes. For 100 notes scored in duplicate by a single evaluator, Cronbach α (calculated by Kuder-Richardson Formula 20 as implemented in R statistical software [version 3.5.1, R Foundation Inc]) was 0.86.

Next, 1 author (D.D.G.) further annotated a random set of 2000 of the 3302 confirmed financial notes in terms of presence or absence of each of 3 features: financial consideration associated with medication treatment (such as nonadherence), financial consideration associated with treatment change (including but not limited to medication), and financial consideration associated with disposition or discharge change (ie, different site or delay in discharge). When the change was not clearly in the context of a financial discussion, based on a reasonable interpretation of the author’s intention as well as proximity in the text, the note was not labeled as including this feature (eg, if a note discussed a patient’s ability to pay and then later indicated a medication change without clearly linking the 2 concepts). These features were not necessarily mutually exclusive, and not all financial notes were so categorized (eg, if a note addressed financial concerns but did not indicate an association with treatment or discharge). An example of financial discussion related to medication is, “She admits to not taking her insulin for 3-4 days…because she could not afford it (was recently laid off from job).” An example of a treatment change incorporating financial consideration is, “He was switched to pravastatin at the time of discharge given its lower cost and his financial concerns and compliance issues.” Likewise, an example of change in discharge is, “…[B]ecause she does not have insurance, she could not have services at home to help with the antibiotic infusions. Therefore the plan was made for her to remain in the hospital.”

### Automated Note Classification

The labeled notes were then used to train random forest classifiers with Python’s scikit-learn package (sklearn.ensemble.RandomForestClassifier, version 1.1.0)^11^ for each of these 3 features. The labeled note corpus was randomly split into a training set of 1600 of the 2000 (80.0%) for model development and tuning and a testing set of 400 (20.0%) used only to characterize model performance. We first applied standard methods in natural language processing to each note, removing punctuation and English-language stop words (common short words such as *a*, *the*, *at*, and *which*); stemming (eliminating suffixes, so that *removing*, *removed*, and *removal* all become *remov*); and generating unigrams and bigrams (single words and 2-adjacent-word combinations). A term frequency–inverse document frequency matrix was created from this set of single words and 2-word combinations, with principal components analysis used to reduce the number of features. This common natural language processing strategy for understanding term importance examines the frequency with which a term appears in a document and the number of documents in which the term appears. The number of principal components was treated as a hyperparameter of the model to be tuned on the training set. (For a general review of natural language processing approaches applied in a clinical context, see Yim et al^12^). Area under the curve calculated by using the trapezoidal rule was used as the primary evaluation metric for discrimination on the training and test sets. For all financial notes, area under the curve in the independent testing set exceeded 0.97; for individual subcategories, area under the curve exceeded 0.89.

### Statistical Analysis

Summary statistics, including proportion for categorical features and mean (SD) for continuous features, were computed for each group (ie, admissions with and admissions without financial notes) using Python’s *pandas* and *scipy* package. No data were missing. Comparisons used χ^2^ tests or single-sample, unpaired, 2-tailed *t* tests, as appropriate. After these univariate analyses, binomial logistic regression models using R’s *glm*, version 3.5.1, adjusted for age, sex, marital status, length of stay, and insurance type at index admission, were fit with presence or absence of financial discussion as the dependent variable to estimate the independent effect of each sociodemographic or clinical feature in terms of odds ratios (ORs) and 95% CIs. All reported *P* values are 2-sided, with nominal significance considered to be uncorrected *P* = .05.

## Results

The corpus as a whole included 1 185 859 notes, reflecting 46 146 index ICU admissions between June 1, 2001, and October 31, 2012; counts for all admissions and notes can be found in eTable 1 in the Supplement. A total of 1936 of 46 146 patients (4.2%) had at least 1 note reflecting financial considerations during their ICU stay. Of these 1936 patients, 1135 (58.6%) were male; the mean (SD) age was 38.8 (28.4) years and mean (SD) length of stay of 21.7 (27.1) days. Among the remaining 44 210 admissions in the cohort, 24 780 (56.1%) were male, mean (SD) age was 48.6 (32.1) years, and mean (SD) length of stay was 9.2 (11.4) days (Table 1).

**Table 1.** Sociodemographic and Clinical Features Associated With Presence or Absence of Financial Notes on 46 146 Index Admissions^a^

Characteristic	No. (%)		*t* Value	*P* Value
	≥1 Financial Note (n = 1936)	No Financial Notes (n = 44 210)		
Age, mean (SD), y	38.8 (28.4)	48.6 (32.1)	−16.8	<.001
Length of stay, mean (SD), d	21.7 (27.1)	9.2 (11.4)	29.4	<.001
			**χ^2^**	***P* Value**
Male sex	1135 (58.6)	24 780 (56.1)	4.89	.03
Race/ethnicity				
White	1248 (62.6)	38 643 (70.1)	50.69	<.001
Black	217 (10.9)	4856 (8.8)	10.02	.002
Unknown or not specified	132 (6.6)	4329 (7.8)	3.87	.049
Hispanic or Latino	110 (5.5)	1519 (2.8)	52.04	<.001
Asian	72 (3.6)	1354 (2.5)	10.10	.001
Other	72 (3.6)	1403 (2.5)	8.29	.004
Insurance				
Private	838 (42.0)	21 142 (38.3)	10.93	.001
Medicare	440 (22.1)	27 020 (49.0)	557.81	<.001
Medicaid	434 (21.8)	4972 (9.0)	363.79	<.001
Other government insurance	201 (10.1)	1498 (2.7)	359.24	<.001
Self-pay	80 (4.0)	520 (0.9)	171.55	<.001
Marital status				
Single	626 (31.4)	11 952 (21.7)	105.43	<.001
Married	516 (25.9)	23 144 (42.0)	204.58	<.001
Divorced	114 (5.7)	2986 (5.4)	0.29	.59
Widowed	106 (5.3)	6962 (12.6)	94.13	<.001
Language				
English	920 (46.1)	27 026 (49.0)	6.20	.01
Spanish	66 (3.3)	968 (1.8)	25.32	<.001
Admission type				
Emergency	1373 (68.9)	39 335 (71.3)	5.58	.02
Newborn	504 (25.3)	7118 (12.9)	253.69	<.001
Elective	75 (3.8)	7417 (13.4)	157.67	<.001
Urgent	40 (2.0)	1281 (2.3)	0.72	.40
Discharge location				
Home	676 (33.9)	17 546 (31.8)	3.77	.05
Home health care	501 (25.2)	13 157 (23.9)	1.64	.20
Rehabilitation/distinct part of hospital	245 (12.3)	6034 (10.9)	3.43	.06
Short-term hospital	135 (6.8)	1373 (2.5)	135.61	<.001
Skilled nursing facility	124 (6.2)	7416 (13.4)	87.14	<.001
Long-term care hospital	107 (5.4)	2097 (3.8)	12.28	<.001
Died in hospital	97 (4.9)	5481 (9.9)	55.66	<.001

^a^ Category percentages do not sum to 100% because rarer groups, including unable to obtain and declined to answer, were omitted for clarity.

In univariate contrasts, patients with financial considerations were, on average, significantly younger (mean [SD] age, 38.8 [28.4] vs 48.6 [32.1] years; *P* < .001) and had longer hospital stays (mean [SD], 21.7 [27.1] vs 9.2 [11.4] days; *P* < .001) (Table 1). Financial notes were also more likely among individuals with Medicaid (434 of 1936 patients [21.8%] vs 4972 of 44 210 [9.0%]; *P* < .001), other non-Medicare government insurance (201 [10.1%] vs 1498 [2.7%]; *P* < .001), and self-pay (80 [4.0%] vs 520 [0.9%]; *P* < .001); those of nonwhite race/ethnicity (among black patients, 217 [10.9%] vs 4856 [8.8%]; *P* = .002); and those who were single (626 [31.4%] vs 11 952 [21.7%]; *P* < .001).

To identify the independent association of these features, logistic regression models were fitted with adjustment for age, sex, race/ethnicity, marital status, insurance category, and length of stay. Table 2 reports resulting ORs for presence of at least 1 financial note during an admission. In particular, robust effects of insurance type (eg, for patients with Medicare insurance: OR, 0.15; 95% CI, 0.08-0.26; *P* < .001), marital status (for single patients: OR, 1.95; 95% CI, 1.51-2.51; *P* < .001), and length of hospital stay (OR, 1.01; 95% CI, 1.01-1.01; *P* < .001) persisted, but age was no longer significantly associated with the presence of financial notes (OR, 1.00; 95% CI, 1.00-1.01; *P* = .07).

**Table 2.** Association Between Sociodemographic and Clinical Features and Presence of Financial Notes in 3565 ICU Patients

Feature	Odds Ratio (95% CI)	*P* Value
Insurance		
Medicare	0.15 (0.08-0.26)	<.001
Private	0.22 (0.13-0.38)	<.001
Marital status, single	1.95 (1.51-2.51)	<.001
Race/ethnicity		
Hispanic or Latino	1.48 (0.89-2.45)	.13
White	1.07 (0.73-1.58)	.72
Male sex	1.04 (0.90-1.21)	.56
Age, y	1.00 (1.00-1.01)	.07
Length of stay, d	1.01 (1.01-1.01)	<.001

Subsequent analysis sought to characterize the documented implications of the financial discussion. Among the 46 146 index admissions, 303 (0.7%) included a discussion of a medication change or the association of financial barriers with previous nonadherence to medication, 142 (0.3%) indicated an associated change in the treatment plan, and 142 (0.3%) included an associated change in the discharge plan. Distributions among subcategories generally reflected a similar distribution of the financial notes as a whole (eTables 2-4 in the Supplement), with the exception that financial notes associated with a treatment change were more common among older than among younger patients (mean [SD] age, 55.1 [20.4] vs 47.9 [32.2] years).

## Discussion

In this investigation of a large corpus of narrative clinical notes drawn from electronic health records, we applied machine learning to develop classifiers able to identify financial discussions in clinical notes, achieving a highly discriminative model. We had expected to find little, if any, evidence of cost consideration in the ICU notes, reflecting the standard argument that such care is high stakes and driven by urgency rather than economic considerations.^1^ A common argument against the feasibility and value of health consumerism claims that the acuity of many health care encounters precludes patient consideration of cost.^1^ By extension, we expected little focus on cost in the ICU given the high likelihood that patients and families were distressed. Furthermore, we recognized that because explicit conversations about cost in clinical encounters are not the norm, any documentation would likely be scant.

We found that 4.2% of ICU admissions included documentation of at least 1 financial consideration and that such discussions were associated with changes in treatment or hospital stay in 0.3% to 0.7% of admissions. These rates are difficult to compare directly to previous work because few estimates of the prevalence of financial conversations exist and none that we could identify in the ICU setting. An analysis of transcribed patient-physician conversations from outpatient visits suggested that approximately 1 in 3 conversations, depending on specialty, included cost discussion.^4^ Some physicians’ associations have advocated for explicit consideration of costs,^7^ and a survey of 1379 oncologists reported that 84% did take patient out-of-pocket costs into consideration.^5^

Beyond clinician consideration of cost, a growing body of evidence suggests that patients desire to engage in cost conversations with their clinicians. In one large study, half of respondents reported having sought cost information before getting care, and 70% reported believing doctors and their staff should discuss cost with patients before ordering services. However, 28% of those surveyed had had a physician or their staff bring up cost.^13^ In another survey, of the 13% of respondents who had actively searched for out-of-pocket costs before seeking care, 63% had called their clinician for information.^14^ Finally, a study of consumer behavior regarding price shopping found that 25% of survey respondents reported discussing costs with their clinician.^15^

### Limitations

We note several important caveats in interpreting these results. First, they are almost certainly underestimates of real-world conversations, which may not be reflected in clinical documentation; for example, a resident physician’s note may not have fully or reliably captured a conversation on rounds. As such, they must be considered simply as establishing minima for prevalence of these conversations. Moreover, it is entirely possible that non-ICU narrative notes (ie, in settings with lesser severity) reflect more financial discussion, and we present our results in the hope that others will investigate diverse settings. Second, our reliance on classifiers to identify types of conversations, which were necessary given the size of the corpus, likely introduced additional uncertainty and the potential for bias. Third, although we identify associations with features such as length of stay, we cannot conclude that such relationships are causal; indeed, in the case of length of stay, the association is likely to be bidirectional, such that length of stay might precipitate a discussion of insurance coverage rather than coverage necessarily influencing length of stay.

## Conclusions

This study represents a necessary first step in understanding how frequently financial considerations explicitly alter care in hospital settings and how such considerations may be associated with sociodemographic features. The results suggest the need for more systematic investigation of such conversations in other contexts to better understand the nature of these considerations. Further study will also be necessary to investigate the extent to which patients and families participate in these conversations, a factor that typically is not reflected in clinical documentation.

## Notes

Supplement. **eTable 1.** Prevalence of Financial Notes as a Proportion of Patients and Individual Notes **eTable 2.** Sociodemographic and Clinical Features Associated With Presence of Financial Notes Reflecting Medication Change **eTable 3.** Sociodemographic and Clinical Features Associated With Presence of Financial Notes Addressing Treatment Plan Change **eTable 4.** Sociodemographic and Clinical Features Associated With Presence of Financial Notes Addressing Discharge Plan Change
