# Supplementary material for: Prevalence and Nature of Financial Considerations Documented in Narrative Clinical Records in Intensive Care Units
Source: JAMA Netw Open. 2018 Nov 2;1(7):e184178. doi: 10.1001/jamanetworkopen.2018.4178 (PMC6324587; doi:10.1001/jamanetworkopen.2018.4178)
Supplement: Supplement. — eTable 1. Prevalence of Financial Notes as a Proportion of Patients and Individual Notes eTable 2. Sociodemographic and Clinical Features Associated With Presence of Financial Notes Reflecting Medication Change eTable 3. Sociodemographic and Clinical Features Associated With Presence of Financial Notes Addressing Treatment Plan Change eTable 4. Sociodemographic and Clinical Features Associated With Presence of Financial Notes Addressing Discharge Plan Change [file jamanetwopen-1-e184178-s001.pdf]

Supplementary Online Content

Gordon DD, Patel I, Pellegrini AM, Perlis RH. Prevalence and nature of financial considerations documented in narrative clinical records in intensive care units. *JAMA Netw Open*. 2018;1(7):e184178. doi:10.1001/jamanetworkopen.2018.4178

eTable 1. Prevalence of Financial Notes as a Proportion of Patients and Individual Notes

eTable 2. Sociodemographic and Clinical Features Associated With Presence of Financial Notes Reflecting Medication Change

eTable 3. Sociodemographic and Clinical Features Associated With Presence of Financial Notes Addressing Treatment Plan Change

eTable 4. Sociodemographic and Clinical Features Associated With Presence of Financial Notes Addressing Discharge Plan Change

This supplementary material has been provided by the authors to give readers additional information about their work.

eTable 1. Prevalence of financial notes as a proportion of patients and individual notes

	**Index admissions = patients (% of patients)**	**All admissions (% of admissions)**	**All notes (% of notes)**
Total N	46,146	58,362	2,083,180
Financial*	1,936 (4.20%)	1,994 (3.42%)	3,492 (0.17%)
Change in Discharge	142 (0.31%)	142 (0.24%)	197 (0.01%)
Change in Treatment	142 (0.31%)	144 (0.25%)	182 (0.01%)
Medication-related	303 (0.66%)	312 (0.53%)	486 (0.02%)

* subcategories of financial notes do not sum to total, as financial notes need not be labeled as belonging to one of these categories

eTable 2. Sociodemographic and clinical features associated with presence of financial notes reflecting medication change				
**Feature**	**1+ Financial/medication-related note**	**No Financial/medication-related note**		
	**Mean (SD)**	**Mean (SD)**	**Student's T**	**P-Value**
Age (years)	47.41 (25.2)	47.90 (32.2)	0.31	0.76
Length of stay (days)	16.97 (25.5)	9.64 (12.4)	4.73	<0.001
	**n (%)**	**n (%)**	**Chi Square**	**P-Value**
Sex (Female)	141 (46.5%)	20090 (43.8%)	0.79	0.374
**Race/Ethnicity**				
White	193 (61.9%)	40293 (69.8%)	8.85	0.003**
Black/African American	51 (16.3%)	5211 (9.0%)	19.3	<0.001
Unknown/Not Specified	17 (5.4%)	4465 (7.7%)	1.96	0.161
Hispanic or Latino	17 (5.4%)	1647 (2.9%)	6.61	0.010*
Asian	6 (1.9%)	1445 (2.5%)	0.22	0.637
Other	7 (2.2%)	1481 (2.6%)	0.03	0.858
**Insurance**				
Private	101 (32.4%)	22111 (38.3%)	4.36	0.037*
Medicare	107 (34.3%)	27775 (48.1%)	23.15	<0.001
Medicaid	61 (19.6%)	5560 (9.6%)	33.81	<0.001
Government	34 (10.9%)	1703 (2.9%)	64.83	<0.001
Self-Pay	8 (2.6%)	593 (1.0%)	5.74	0.017*
**Marital Status**				
Single	110 (35.3%)	12877 (22.3%)	29.25	<0.001
Married	92 (29.5%)	23896 (41.4%)	17.63	<0.001
Divorced	22 (7.1%)	3144 (5.4%)	1.26	0.262
Widowed	30 (9.9%)	7097 (12.3%)	1.82	0.177
**Language**				
English	189 (60.6%)	28358 (49.1%)	15.87	<0.001
Spanish	13 (4.2%)	1042 (1.8%)	8.43	0.004**
**Admission Type**				
Emergency	257 (82.4%)	41246 (71.4%)	17.69	<0.001
Newborn	40 (12.8%)	7602 (13.2%)	0.01	0.924
Elective	9 (2.9%)	7566 (13.1%)	27.67	<0.001
Urgent	5 (1.6%)	1326 (2.3%)	0.39	0.531
**Discharge Location**				

Home	118 (37.8%)	18377 (31.8%)	4.87	0.027*
Home Health Care	103 (33.0%)	13765 (23.8%)	13.87	<0.001
Rehabilitation/Distinct Part Hospital	17 (5.4%)	6377 (11.0%)	9.35	0.002**
Short-Term Hospital	5 (1.6%)	1522 (2.6%)	0.92	0.337
Skilled Nursing Facility	27 (8.7%)	7635 (13.2%)	5.26	0.022*
Long-Term Care Hospital	12 (3.8%)	2267 (3.9%)	0.01	0.941
Died in Hospital	9 (2.9%)	5615 (9.7%)	15.82	<0.001

eTable 3. Sociodemographic and clinical features associated with presence of financial notes addressing treatment plan change

Feature	1+ Financial/treatment-related note	No Financial/treatment-related note		
	**Mean (SD)**	**Mean (SD)**	**Student's T**	**P-Value**
Age (years)	55.13 (20.4)	47.85 (32.2)	5.11	<0.001
Length of stay (days)	13.47 (16.6)	9.66 (12.5)	3.47	0.001
	**n (%)**	**n (%)**	**Chi square**	**P-Value**
Sex (Female)	67 (47.2%)	20164 (43.8%)	0.52	0.472
**Race/Ethnicity**				
White	88 (61.1%)	40458 (69.6%)	4.52	0.034
Black/African American	17 (11.8%)	5366 (9.2%)	0.85	0.357
Unknown/Not Specified	6 (4.2%)	4476 (7.7%)	2.05	0.152
Hispanic or Latino	9 (6.2%)	1668 (2.9%)	4.72	0.030*
Asian	5 (3.5%)	1443 (2.5%)	0.24	0.622
Other	4 (2.8%)	1487 (2.6%)	0.01	0.922
**Insurance**				
Private	44 (30.6%)	22201 (38.2%)	3.24	0.072
Medicare	50 (34.7%)	27911 (48.0%)	9.66	0.002**
Medicaid	32 (22.2%)	5662 (9.7%)	23.97	<0.001
Government	13 (9.0%)	1741 (3.0%)	15.89	<0.001
Self-Pay	4 (2.8%)	599 (1.0%)	2.74	0.098
**Marital Status**				
Single	56 (38.9%)	13051 (22.5%)	21.31	<0.001
Married	49 (34.0%)	23965 (41.2%)	2.79	0.095
Divorced	10 (6.9%)	3182 (5.5%)	0.35	0.555
Widowed	17 (11.8%)	7139 (12.3%)	0	0.962
**Language**				
English	104 (72.2%)	28605 (49.2%)	29.49	<0.001
Spanish	6 (4.2%)	1059 (1.8%)	3.19	0.074
**Admission Type**				
Emergency	121 (84.0%)	41576 (71.5%)	10.4	0.001**
Newborn	5 (3.5%)	7641 (13.1%)	10.96	0.001***
Elective	14 (9.7%)	7567 (13.0%)	1.1	0.293
Urgent	3 (2.1%)	1329 (2.3%)	0.01	0.908
**Discharge Location**				

Home	57 (39.6%)	18520 (31.9%)	3.59	0.058
Home Health Care	47 (32.6%)	13869 (23.9%)	5.61	0.018*
Rehabilitation/Distinct Part Hospital	4 (2.8%)	6407 (11.0%)	9.15	0.002**
Short-Term Hospital	1 (0.7%)	1533 (2.6%)	1.43	0.232
Skilled Nursing Facility	12 (8.3%)	7674 (13.2%)	2.57	0.109
Long-Term Care Hospital	4 (2.8%)	2287 (3.9%)	0.25	0.618
Died in Hospital	6 (4.2%)	5623 (9.7%)	4.38	0.036*

eTable 4. Sociodemographic and clinical features associated with presence of financial notes addressing discharge plan change				
**Feature**	**1+ Financial/discharge-related note**	**No Financial/discharge-related note**		**P-Value**
	**Mean (SD)**	**Mean (SD)**	**Student's T**	
Age (years)	48.46 (23.1)	47.86 (32.2)	1.93	0.056
Length of stay (days)	27.17 (32.2)	9.63 (12.4)	8.3	<0.001
	**n (%)**	**n (%)**	**Chi square**	
Sex (Female)	42 (29.6%)	20189 (43.9%)	11.2	<0.001
**Race/Ethnicity**				
White	94 (66.2%)	40447 (69.6%)	0.63	0.426
Black/African American	13 (9.9%)	5361 (9.2%)	0.01	0.908
Unknown/Not Specified	6 (4.9%)	4473 (7.7%)	1.94	0.163
Hispanic or Latino	3 (2.1%)	1672 (2.9%)	0.09	0.769
Asian	6 (4.2%)	1442 (2.5%)	1.13	0.288
Other	2 (1.4%)	1486 (2.6%)	0.36	0.548
**Insurance**				
Private	55 (39.4%)	22179 (38.2%)	0	0.961
Medicare	25 (17.6%)	27936 (48.1%)	51.52	<0.001
Medicaid	37 (26.1%)	5649 (9.7%)	41.05	<0.001
Government	15 (10.6%)	1737 (3.0%)	25.31	<0.001
Self-Pay	9 (6.3%)	592 (1.0%)	34.2	<0.001
**Marital Status**				
Single	57 (40.1%)	13062 (22.5%)	24.3	<0.001
Married	46 (32.4%)	23953 (41.2%)	4.21	0.040*
Divorced	6 (4.9%)	3178 (5.5%)	0.22	0.64
Widowed	10 (7.0%)	7134 (12.3%)	3.14	0.076
**Language**				
English	75 (52.8%)	28625 (49.3%)	0.58	0.448
Spanish	5 (3.5%)	1059 (1.8%)	1.43	0.232
**Admission Type**				
Emergency	119 (83.8%)	41555 (71.5%)	9.89	0.002**
Newborn	13 (9.2%)	7634 (13.1%)	1.64	0.2
Elective	6 (4.2%)	7576 (13.0%)	8.96	0.003**
Urgent	4 (2.8%)	1328 (2.3%)	0.02	0.887
**Discharge Location**				
Home	44 (31.0%)	18531 (31.9%)	0.02	0.886

Home Health Care	23 (16.2%)	13895 (23.9%)	4.23	0.040*
Rehabilitation/Distinct Part Hospital	33 (23.2%)	6363 (11.0%)	20.63	<0.001
Short-Term Hospital	6 (4.9%)	1526 (2.6%)	0.86	0.354
Skilled Nursing Facility	11 (7.7%)	7669 (13.2%)	3.22	0.073
Long-Term Care Hospital	10 (7.0%)	2276 (3.9%)	2.89	0.089
Died in Hospital	5 (3.5%)	5627 (9.7%)	5.48	0.019*
